# Laparoscopic surgery of benign entero-vesical or entero-vaginal fistulae

**DOI:** 10.1007/s00384-015-2395-3

**Published:** 2015-09-30

**Authors:** Matthias Kraemer, David Kara

**Affiliations:** Abteilung Allgemeine und Viszeralchirurgie, Koloproktologie, St. Barbara-Klinik, Am Heessener Wald 1, 59073 Hamm, Germany

**Keywords:** Laparoscopic surgery, Diverticular disease, Entero-vesical fistulae, Entero-vaginal fistulae, Crohn’s disease

## Abstract

**Purpose:**

Entero-vesical or entero-vaginal fistulae (EVF) are an uncommon septic complication mainly of diverticular disease. The fistulae are usually situated within extensive and dense inflammatory masses occluding the entrance of the pelvis. There are still some controversies regarding laparoscopic feasibility and treatment modalities of this disorder.

**Methods:**

A retrospective chart review of all patients with EVF operated at our department since 2008. Patients were identified by use of the computerized hospital information system.

**Results:**

In nineteen patients (ten males), median age 68 years, 13 patients had entero-vesical fistulae, and 6 patients had entero-vaginal fistulae. The fistulae were caused by complicated diverticular disease in 16 patients (84 %), Crohn’s disease (two patients), and ulcerative colitis (one patient). All cases were attempted laparoscopically. Operative treatment involved separation of the inflammatory mass and resection of the affected colorectal segment. There were three conversions (16 %), all three requiring bladder repair considered too extensive for laparoscopic means. In two further patients small bladder defects were sutured laparoscopically, the remaining patients required no bladder repair. The inferior mesentric artery (IMA) was preserved in all cases. Median operative time was 180 min. Two patients received a protective ileostomy: one converted patient and one cachectic patient with Crohn’s disease under immune-modulating therapy. Both ileostomies were closed. Altogether, there were five complications in five patients (26 %), four of them were minor (Clavien grade I and II). The cachectic patient with Crohn’s disease suffered a major (grade IIIb) complication (stoma prolapse, treated by early closure of the ileostomy). There was no anastomotic leakage and no mortality. Median hospital stay was 12 days.

**Conclusions:**

The laparoscopic approach is a safe option for the treatment of EVF of benign inflammatory origin. In most cases it offers all the advantages pertaining to minimally invasive surgery. For a definite and causal approach, the disorder belongs primarily within the therapeutic domain of the visceral surgeon. Following the separation of the inflammatory colon, most of the bladder lesions caused by EVF will heal without further surgical measures.

## Introduction

Entero-vesical or entero-vaginal fistulae (EVF) are uncommon. The overwhelming majority are of benign inflammatory origin, only about 10 % are caused by malignancies of the bowel or the bladder. The main causes for benign fistula formation are diverticular disease (70 %) and Crohn’s disease (5–10 %) [1–5]. Rarer causes include appendicitis, Meckel diverticulitis, radiotherapy, and trauma.

Fistulae are usually situated within extensive and dense inflammatory masses occluding the entrance of the pelvis. Many surgeons therefore consider this disorder still as technically demanding if not unsuitable for laparoscopic surgery [6]. Up to 2014, only 204 cases of intended laparoscopic repairs of entero-vesical or entero-vaginal fistulae have been reported in 25 publications [7], many of them within larger cohorts of “complicated diverticulitis”, demonstrating that there is still a paucity of specific data. Furthermore, in some places controversies still exist regarding the extent and modalities of treatment and whether such fistulae belong primarily in the domains of urologists or gynaecologists rather than of visceral surgeons [8, 9].

In an attempt to further clarify some of the controversial issues, we report our experiences of 19 consecutive patients who were operated at our institution since 2008.

## Patients and methods

A chart review was conducted of all consecutive patients referred to the department with the diagnosis of entero-vesical or colo-vaginal fistula since October 2008. Patients were identified by use of the computerized hospital information system. Laparoscopic colorectal surgery was introduced to the department in 2004. Prior to 2008, patients with entero-vesical fistula were usually treated by the urology department. In this era, they were operated conventionally by the urologists, and surgical assistance was only sought if deemed necessary.

Usually, patients referred to the department have their diagnosis already confirmed elsewhere. Routine preoperative workup includes endoscopy, barium colonic contrast enema, and CT scan. The main purpose of the preoperative workup is the exclusion of a neoplastic cause for the fistula. Likewise, a cystoscopy is ordered only if a neoplastic lesion of the bladder has to be excluded.

Except for suspected malignancy, all cases are primarily attempted laparoscopically. Colorectal malignancy as cause for the fistula formation is considered as relative contraindication for laparoscopic surgery as oncological considerations usually require excisions of a larger part of the bladder. We believe that in these cases the necessary extent of bladder resection can better be judged by direct tactile sensation. Furthermore, a complex laparoscopic bladder repair is beyond our scope of safe feasibility, in particular if the margin is anywhere near the trigone.

Operative strategy aims initially at separation of the inflammatory mass from the bladder (or the vagina). This is done by blunt instrumental “pinching” or by sharp dissection, as required. Thereafter, the inflammatory mass is mobilized sufficiently to allow for safe dissection of the mesentery. We attempt to preserve the inferior mesenteric artery (IMA) by finding a plane in between the affected colo-rectum and the vasculature. The rectum is mobilized dorsally downwards and if required ventrally beyond the peritoneal flexure until a 33 bougie can be passed easily and without resistance peranally up to the intended aboral resection margin.

If feasible and particularly in younger and fit patients, the entire segment of colon affected by diverticulosis is resected. We believe that even in elderly patients where we would initially aim at only a limited resection it does not make sense to leave colon behind which is grossly altered by diverticular disease. The extent of diverticulosis can best be seen on colonic contrast enema, which we therefore find very useful in our preoperative assessment and planning of the operation.

At the end of the operation, the bladder is filled with saline via a transurethral catheter and checked for leakage. In case of leakage, the bladder defect which is usually minute is closed by some sutures and the tightness of the closure confirmed by a refill of the bladder. The urethral catheter is left for 5 to 7 days and then removed after the bladder integrity is shown by a cystography.

## Results

Thirteen patients with colo-vesical fistulae and six patients with colo-vaginal fistulae were operated during the period under review. Patient demographics are shown in Table 1. The cause for the fistulae was diverticular disease in 16 cases (84 %); two fistulae were caused by Crohn’s disease and one by ulcerative colitis. Operative details are presented in Table 2. Three of the 19 cases were converted (16 %). All three cases were converted since the operating surgeon considered the bladder defect too extensive for a safe laparoscopic repair. Small bladder defects were sutured laparoscopically in two further patients; the remaining patients required no specific bladder repair. Two patients received a protective ileostomy. One was a converted patient; the other was a severely cachectic patient with Crohn’s disease under immune-modulating therapy. Both protective ileostomies could meanwhile be closed. No intraoperative complications were recorded and no perioperative blood transfusions were required. Altogether, five complications were noted in five patients (26 %, Table 3). There were no anastomotic leaks, nor mortality.

**Table 1:** Patient demographics

Gender (male/female)	10/9
Age (yrs. median/range)	68/23–84
Underlying disease	Diverticular disease: 16 (84 %) Crohn’s disease: 2 (11 %) Ulcerative colitis: 1 (5 %)
Type of fistula	Colo-vesical 13 Colo-vaginal 6

**Table 2:** Perioperative details

Conversions	3 (16 %)
Median operative time (min)	180 (72–355)
Intraoperative complications	0
Protective ileostomy	2 (11 %)
Blood transfusions	0
Median hospital stay (days)	12 (9–25)

**Table 3:** Complications

	*n*	
Minor	4 (21 %)	
Clavien grade I (requiring no intervention)	2	Transient diarrhoea
		Transient anal bleeding
Clavien grade II (requiring non-operative intervention)	2	Pneumonia
		Persisting urinary tract infection
Major	1 (5 %)	
Clavien III b (requiring operative intervention under GA)	1	Ileostomy prolapse in cachectic patient with Crohn’s disease; treated by early closure of ileostomy; uneventful further course

## Discussion

This study confirms results of the other published series: the laparoscopic approach for entero-vesical or entero-vaginal fistula of benign inflammatory origin is safe and offers in most cases all the advantages pertaining to minimally invasive surgery: in particular low morbidity and rapid recovery [7, 10–17]. Conversion rates are low, ranging from 6 to 36 % (Table 4). Furthermore, converted cases do not fare worse than one would expect if these patients were operated conventionally [7]. Morbidity and mortality is similar to laparoscopic surgery of uncomplicated diverticular disease [7].

**Table 4:** Published series of laparoscopic surgery for entero-vesical/vaginal fistulae

Author	Study period	*n*	Conversions	Operative time median/mean/range	Complications		Mortality	Division of IMA	Bladder repair	Diverting stoma
					minor	major				
Menenakos [10]	1993–2003	18	1 (6 %)	n.s./237/165–330	3 (20 %)	2 (13 %)	0	yes	6 (40 %)	0
Nguyen [11]	1994–2004	14	5 (36 %)	n.s./209/78–309	2 (14 %)	0	0	n.s.	3 (38 %)	
Engledow [12]	1995–2005	31	9 (29 %)*	150/n.s./60–310	1 (3 %)	3 (10 %)	2 (6 %)	yes	2 (9 %)	1 (3 %)
Mizushima [14]	n.s.	4	0	234/223/100–325	1 (25 %)	0	0	n.s.	1 (25 %)	0
Abbass [16]	2006–2012	21	0	240/254/168–360	5 (24 %)	3 (14 %)	0	n.s.	n.s.	1 (5 %)
Marney [17]	2004–2011	15	5 (33 %)	135/n.s./85–240	3 (20 %)	0	0	yes	n.s.	0
own results	2008–2014	19	3 (16 %)	180/176/72–355	4 (21 %)	1 (5 %)	0	no	3 (16 %)	2 (11 %)

*n.s*. not stated

*10 % since 2000

Considering the overall safety of the procedure it remains puzzling why some authors still propose a “conservative approach”, which means simply dividing the fistula and leaving the diseased colon behind [18–22]. This overtly hesitant approach is without any apparent advantage for the patient but carries a considerable risk of further aggravation and spread of the inflammatory disease process later on.

In this context, it is important to point out that in the overwhelming majority of cases entero-vesical fistulae are manifestations of a surgical disease of the bowel (not of the bladder) and that the proper causal therapy of this disorder therefore mandates a visceral surgical approach. Fistulae have a high pressure gradient from the colon to the bladder/vagina, not vice versa. Patients therefore typically complain of the passage of gas or faecal matter via the urethra (or vagina). That urine seeps the other way is very rare. If found, it is typically caused by concomitant urinary retention such as produced by prostatic hypertrophy. A simple separation of the diseased colon as cause of the high pressure blow hole usually suffices to heal the bladder. Further repairs of the bladder are usually not required [11, 12, 14]. Only larger defects need to be repaired. Therefore, for a definite and causal approach, the disorder belongs primarily within the therapeutic domain of the visceral surgeon. Even more so if patients want to profit from the potential benefits of a minimally invasive approach.

It is of interest and has previously been noted that entero-vesical fistulae are predominantly found in males and only very rarely encountered in non-hysterectomised females [2, 5, 9]. This was also confirmed in our series with only three female patients with entero-vesical fistula, all of them hysterectomised. It appears that the uterus protects the bladder anatomically from inflammatory fistula formation by diverticular disease.

On a more technical note, some surgeons routinely dissect and ligate the IMA as part of the procedure [12, 13, 15, 17]. We believe that this is unnecessary and unwarranted in this benign disease scenario and that by simply dissecting the mesenteric plane in between the proper bowel and the vasculature the artery and therefore a potentially beneficial extra of blood supply to the rectum can be retained in the majority of cases without much additional effort.
